# Different inflammatory blood markers correlate with specific outcomes in incident HPV-negative head and neck squamous cell carcinoma: a retrospective cohort study

**DOI:** 10.1186/s12885-022-09327-4

**Published:** 2022-03-05

**Authors:** Paolo Boscolo-Rizzo, Andrea D’Alessandro, Jerry Polesel, Daniele Borsetto, Margherita Tofanelli, Alberto Deganello, Michele Tomasoni, Piero Nicolai, Paolo Bossi, Giacomo Spinato, Anna Menegaldo, Andrea Ciorba, Stefano Pelucchi, Chiara Bianchini, Diego Cazzador, Giulia Ramaciotti, Valentina Lupato, Vittorio Giacomarra, Gabriele Molteni, Daniele Marchioni, Cristoforo Fabbris, Antonio Occhini, Giulia Bertino, Jonathan Fussey, Giancarlo Tirelli

**Affiliations:** 1 Department of Medical, Surgical and Health Sciences, Section of Otolaryngology, University of Trieste, Trieste, Italy; 2 Unit of Cancer Epidemiology, Centro di Riferimento Oncologico di Aviano (CRO) IRCCS, Aviano, Italy; 3 Department of ENT, Addenbrooke’s Hospital, Cambridge University Hospitals NHS Foundation Trust, Cambridge, UK; 4 Department of Otolaryngology, Head and Neck Surgery, University of Brescia, Brescia, Italy; 5 Department of Medical Oncology, University of Brescia, Brescia, Italy; 6 Unit of Otolaryngology, AULSS 2 - Marca Trevigiana, Treviso, Italy; 7 ENT Department, University Hospital of Ferrara, Ferrara, Italy; 8 Department of Neurosciences, Section of Otolaryngology, University of Padova, Padova, Italy; 9 Unit of Otolaryngology, Azienda Ospedaliera “S. Maria Degli Angeli”, Pordenone, Italy; 10 Department of Surgical Sciences, Dentistry, Gynecology and Pediatrics, Section of Otolaryngology, University of Verona, Verona, Italy; 11 Department of Otolaryngology, University of Pavia, IRCCS Policlinico San Matteo Foundation, Pavia, Italy; 12 Department of ENT/Head and Neck Surgery, Elizabeth University Hospital Birmingham, Birmingham, Queen UK

**Keywords:** Head and neck cancer, Blood markers, Inflammatory system, Overall survival, Local recurrence

## Abstract

**Background:**

Inflammatory blood markers have been associated with oncological outcomes in several cancers, but evidence for head and neck squamous cell carcinoma (HNSCC) is scanty. Therefore, this study aims at investigating the association between five different inflammatory blood markers and several oncological outcomes.

**Methods:**

This multi-centre retrospective analysis included 925 consecutive patients with primary HPV-negative HNSCC (median age: 68 years) diagnosed between April 2004 and June 2018, whose pre-treatment blood parameters were available. Neutrophil to lymphocyte ratio (NLR), platelet to lymphocyte ratio (PLR), lymphocyte to monocyte ratio (LMR), systemic inflammatory marker (SIM), and systemic immune-inflammation index (SII) were calculated; their associations with local, regional, and distant failure, disease-free survival (DFS), and overall survival (OS) was calculated.

**Results:**

The median follow-up was 53 months. All five indexes were significantly associated with OS; the highest accuracy in predicting patients’ survival was found for SIM (10-year OS = 53.2% for SIM < 1.40 and 40.9% for SIM ≥ 2.46; c-index = 0.569) and LMR (10-year OS = 60.4% for LMR ≥ 3.76 and 40.5% for LMR < 2.92; c-index = 0.568). While LMR showed the strongest association with local failure (HR = 2.16; 95% CI:1.22–3.84), PLR showed the strongest association with regional (HR = 1.98; 95% CI:1.24–3.15) and distant failure (HR = 1.67; 95% CI:1.08–2.58).

**Conclusion:**

Different inflammatory blood markers may be useful to identify patients at risk of local, regional, or distant recurrences who may benefit from treatment intensification or intensive surveillance programs.

**Supplementary Information:**

The online version contains supplementary material available at 10.1186/s12885-022-09327-4.

## Background

Despite the strong evidence that host-related factors can significantly influence outcomes, the estimation of prognosis in cancer patients is still essentially based only on tumour parameters incorporated in the TNM staging system, evaluated by means of clinical and histopathological assessment and on performance status, judged on patient’s functionality [1]. Therefore, in current clinical practice there is still little consideration and appreciation of the prognostic potential of host-related factors.

In recent years, among the host-related factors that have aroused considerable interest, those based on the evaluation of blood parameters, in particular pre-treatment peripheral blood leukocytes, showed promising prognostic correlations [2, 3]. These indices aim to characterise the inflammatory status of the patient and embrace the concept of cancer as a systemic inflammatory disease. There is indeed increasing evidence that tumour-associated inflammation plays an important role in the development, progression and metastasis of cancer and might be related to systemic inflammation [4].

Head and neck squamous cell carcinoma (HNSCC) is the sixth commonest cancer worldwide, with 890,000 new cases documented every year, and it is the seventh commonest cause of cancer-related death, with a 40–50% mortality [5]. Human papillomavirus (HPV) is a validated and robust biomarker, but its utility is however limited to oropharyngeal squamous cell carcinoma [6]. Therefore, other valuable prognostic biomarkers are needed for HPV–negative HNSCC.

HNSCC promotes local and systemic inflammation [6]. Thus, host factors indicating inflammatory status have also been investigated in these malignancies. Several indices have been proposed, the most of them combining routinary blood parameters such as neutrophils, lymphocytes, monocytes, and platelets count [7–16]. The aim of this study was to evaluate and compare the five inflammatory blood markers (IMBs) with respect to different clinical endpoints using pre-treatment blood tests in a large series of patients receiving upfront surgery for HNSCCs.

## Methods

### Inclusion criteria

This multi-centre retrospective study was performed in a cohort of consecutive patients diagnosed with primary HNSCC from April 1, 2004 to June 30, 2018, who underwent upfront surgery with/without adjuvant (chemo)radiotherapy. The study network included General and University Hospitals in North Italy, located in Brescia, Ferrara, Padova, Pavia, Pordenone, Treviso, Trieste, and Verona. Inclusion criteria were: (a) HNSCC arising from the oral cavity, oropharynx, hypopharynx, or larynx; (b) curative upfront surgery as primary treatment modality; and (c) availability of pre-operative neutrophils, lymphocytes, monocytes, and platelets count, i.e., the blood parameters necessary for the calculation of the inflammatory blood markers under investigation. Patients were specifically excluded if: (a) they were diagnosed with nasopharyngeal carcinoma or T1 glottic squamous cell carcinoma; (b) they had any coexisting conditions or haematological conditions that could alter inflammatory parameters; (c) they had previous malignancy or additional synchronous primary tumours; (d) their pre-treatment blood test results were not available; (e) they had metastatic disease; and (f) they had HPV-positive disease.

### Participants and data

Medical records were reviewed to collect socio-demographic and clinical characteristics of enrolled patients. Baseline characteristics, including gender, age, smoking habits, drinking habits, cancer site, clinical and pathological TNM staging (7^th^ edition), grading, surgical margins and extranodal extension were retrieved. For oropharyngeal carcinomas, HPV status was assessed by p16 immunostaining and/or HPV-PCR. Blood parameters collected at baseline were platelet (10^3^/μL), haemoglobin (Hb, g/L), neutrophils (10^3^/μL), lymphocytes (10^3^/μL), monocytes (10^3^/μL). Patients were routinely followed-up according to consensus guidelines [17] with endoscopic examination of the upper aero-digestive tract every one-to-three months for the first year, three-to-four months during the second year, four-to-six months during the third year, and every six months thereafter. A chest computed tomography scan was annually performed in patients with history of smoking ≥ 20 pack/year. Additional dedicated head and neck imaging was acquired based on clinical features and local protocol. No patient was lost to follow-up.

### Inflammatory blood markers

Using pre-treatment blood parameters, we investigated five pre-treatment indexes: 1) neutrophil to lymphocyte ratio (NLR) [7–10], calculated as NLR = neutrophils / lymphocytes; 2) platelet to lymphocyte ratio (PLR) [11–13], calculated as PLR = platelets / lymphocytes; 3) lymphocyte to monocyte ratio (LMR) [14], calculated as LMR = lymphocytes / monocytes; 4) systemic inflammatory marker (SIM) [15], calculated as SIM = [neutrophils X monocytes] / lymphocytes; 5) systemic immune-inflammation index (SII) [16], calculated as SII = [neutrophils X platelets] / lymphocytes.

### Statistics

For each patient, the time at risk was computed from the date of surgery to the event date or last follow-up, whichever occurred first according to the outcome of interest. The event of interest was defined as: death from any cause for overall survival (OS); disease recurrence or death from any cause for disease-free survival (DFS); local recurrence for local failure; regional recurrence for regional failure; distant metastasis for distant failure. The Kaplan–Meier method was used to generate crude survival probabilities and the log-rank test was used to assess the heterogeneity in time to event according to strata of selected covariates [18]. To account for competing risks, local, regional, and distant failures were evaluated through cumulative incidence [19], and differences according to blood parameters were tested through Gray's test [20]. Hazard ratios (HR) and the corresponding 95% CI were calculated using Cox proportional hazards models [18], adjusting for study centre, gender, and age, plus clinically relevant covariates (i.e., pT, pN, surgical margins, extranodal extension, adjuvant [chemo]radiotherapy). For local regional, and distant recurrence, risk estimates were adjusted for competing risk according to the Fine-Gray model [19]. Blood parameters and inflammatory indexes were categorized in three levels; the optimal cut-offs were determined according to a recursive algorithm that maximizes the model predictability in OS, measured through Harrell’s C-index [21]. Haemoglobin level was categorized as low, normal or high according to gender-specific clinical cut-offs (i.e., < 12 g/L, 12–16 g/dL, and > 16 g/dL in women; < 14 g/dL, 14–18 g/dL, and > 18 g/dL in men).

## Results

### Population

Out of 1001 eligible patients, 925 patients were included the present analysis (median age: 68 years; interquartile range: 61–76 years). The majority of patients (*n* = 679, 73.4%) were male, with stage III–IV cancer (*n* = 642, 69.4%) and with moderately differentiated SSC (*n* = 469, 50.7%; Table 1). Negative surgical margins were achieved in 610 patients (65.9%) and extranodal extension was absent in 765 patients (82.7%). Adjuvant (chemo)radiotherapy was administered to 463 patients (50.1%). During a median follow-up of 53 months (interquartile range: 31–82 months), 385 patients died; cancer was the cause of death in 215 (55.8%) of them. One hundred forty-one patients experienced local recurrence, while 127 patients had regional recurrence and 111 distant metastases. A second primary head and neck or lung tumour was diagnosed during follow-up in 72 patients. Lower OS was associated with increased age (HR = 2.28; 95% CI: 1.64–3.18 for age 70–79 years and HR = 3.82; 95% CI: 2.64–5.54 for age > 80 years), more advanced pT stage (HR = 1.90; 95% CI: 1.18–3.08 for pT3 and HR = 2.52; 95% CI: 1.58–4.02 for pT4), more advanced pN stage (HR = 1.72; 95% CI: 1.23–2.40 for pN1 and HR = 2.12; 95% CI: 1.51–2.97 for pN2-pN3), close/positive surgical margins (HR = 1.27; 95% CI: 1.00–1.62), and extranodal extension (HR = 1.41; 95% CI: 1.04–1.92). Similar patterns were found for DFS (Table 1).

**Table 1:** Risk of recurrence and death according to socio-demographic and clinical characteristics

	Patients	Disease-free survival			Overall survival		
		Events	(%)	HR (95% CI)	Events	(%)	HR (95% CI)
Gender							
Female	246	112	(45.5)	Ref	93	(37.8)	Ref
Male	679	334	(49.2)	1.07 (0.85–1.35)	292	(43.0)	1.15 (0.89–1.48)
Age (years)							
< 60	195	70	(35.9)	Ref	52	(26.7)	Ref
60–69	312	137	(43.9)	1.32 (0.98–1.77)	115	(36.9)	1.55 (1.11–2.17)
70–79	281	143	(50.9)	1.77 (1.31–2.37)	132	(47.0)	2.28 (1.64–3.18)
≥ 80	137	96	(70.1)	2.99 (2.14–4.16)	86	(62.8)	3.82 (2.64–5.54)
Smoking habits							
Never	178	81	(45.5)	Ref	69	(38.8)	Ref
Ever	655	333	(50.8)	1.33 (1.01–1.44)	279	(42.6)	1.35 (1.00–1.83)
* Missing*	*92*	*42*	*(46.6)*		*37*	*(40.2)*	-
Drinking habits							
Never	453	219	(48.3)	Ref	183	(40.4)	Ref
Ever	319	157	(49.2)	1.14 (0.90–1.43)	138	(43.3)	1.24 (0.97–1.59)
*Missing*	153	*70*	*(45.8)*		64	(41.8)	-
Cancer site							
Oral cavity	413	199	(48.2)	1.31 (0.92–1.87)	175	(42.4)	1.69 (1.13–2.54)
Oropharynx	93	43	(46.2)	Ref	32	(34.4)	Ref
Hypopharynx	60	36	(60.0)	1.60 (0.99–2.56)	33	(55.0)	1.84 (1.10–3.10)
Larynx	359	168	(46.8)	1.26 (0.87–1.81)	145	(40.4)	1.52 (1.00–2.30)
pT							
pT1	93	35	(37.6)	Ref	24	(25.8)	Ref
pT2	320	145	(45.3)	1.31 (0.89–1.93)	122	(38.1)	1.71 (1.09–2.71)
pT3	220	103	(46.8)	1.55 (1.03–2.34)	86	(39.1)	1.90 (1.18–3.08)
pT4	282	158	(56.0)	1.83 (1.23–2.72)	148	(52.5)	2.52 (1.58–4.02)
* Missing*	*10*	5	(50.0)		*5*	*(50.0)*	*-*
pN							
pN0	534	212	(39.7)	Ref	138	(32.5)	Ref
pN1	121	68	(56.2)	1.67 (1.23–2.26)	59	(49.6)	1.72 (1.23–2.40)
pN2-pN3	265	162	(61.1)	1.93 (1.42–2.64)	144	(56.1)	2.12 (1.51–2.97)
* Missing*	*5*	4	(80.0)		*3*	*(60.0)*	*-*
Grading							
G1	93	37	(39.8)	Ref	32	(34.4)	Ref
G2	469	211	(45.0)	1.30 (0.90–1.77)	180	(38.4)	1.21 (0.81–1.80)
G3	303	166	(54.8)	1.43 (0.97–2.12)	144	(47.5)	1.32 (0.87–2.01)
* Missing*	*60*	*32*	*(53.3)*		*29*	*(48.3)*	
Surgical margins							
Negative	610	263	(43.1)	Ref	224	(36.7)	Ref
Close/positive	243	140	(57.6)	1.31 (1.05–1.63)	120	(49.4)	1.27 (1.00–1.62)
* Missing*	*72*	*43*	*(59.7)*		*41*	*(56.9)*	
Extranodal extension							
Absent	765	336	(43.9)	Ref	283	(37.0)	Ref
Present	160	110	(68.8)	1.34 (1.00–1.79)	102	(63.8)	1.41 (1.04–1.92)
Adjuvant (chemo) radiotherapy							
No	454	198	(43.6)	Ref	164	(36.1)	Ref
Yes	463	241	(52.1)	0.76 (0.58–1.01)	214	(46.2)	0.81 (0.60–1.10)
* Missing*	*8*	*7*	*(87.5)*		*7*	*(87.5)*	

Hazard ratio (*HR*) and corresponding 95% confidence intervals (*CI*) were estimated through Cox proportional hazard model, adjusting for study centre, gender, age, cancer site, pT, pN, surgical margins, extranodal extension, and adjuvant (chemo) radiotherapy

Blood samples were obtained at a median of 19 days days before surgery (interquartile range: 10–31 days). Supplementary Table 1 shows the association between blood parameters and patient outcomes. Low haemoglobin levels were significantly associated with lower DFS (HR = 1.35, 95% CI: 1.10–1.68) and OS (HR = 1.56, 95% CI 1.24–1.95). Moreover, lymphocyte count < 1.97 10^3^/μL was associated with a reduction of DFS and OS.

### Inflammatory blood markers

**Fig. 1:**
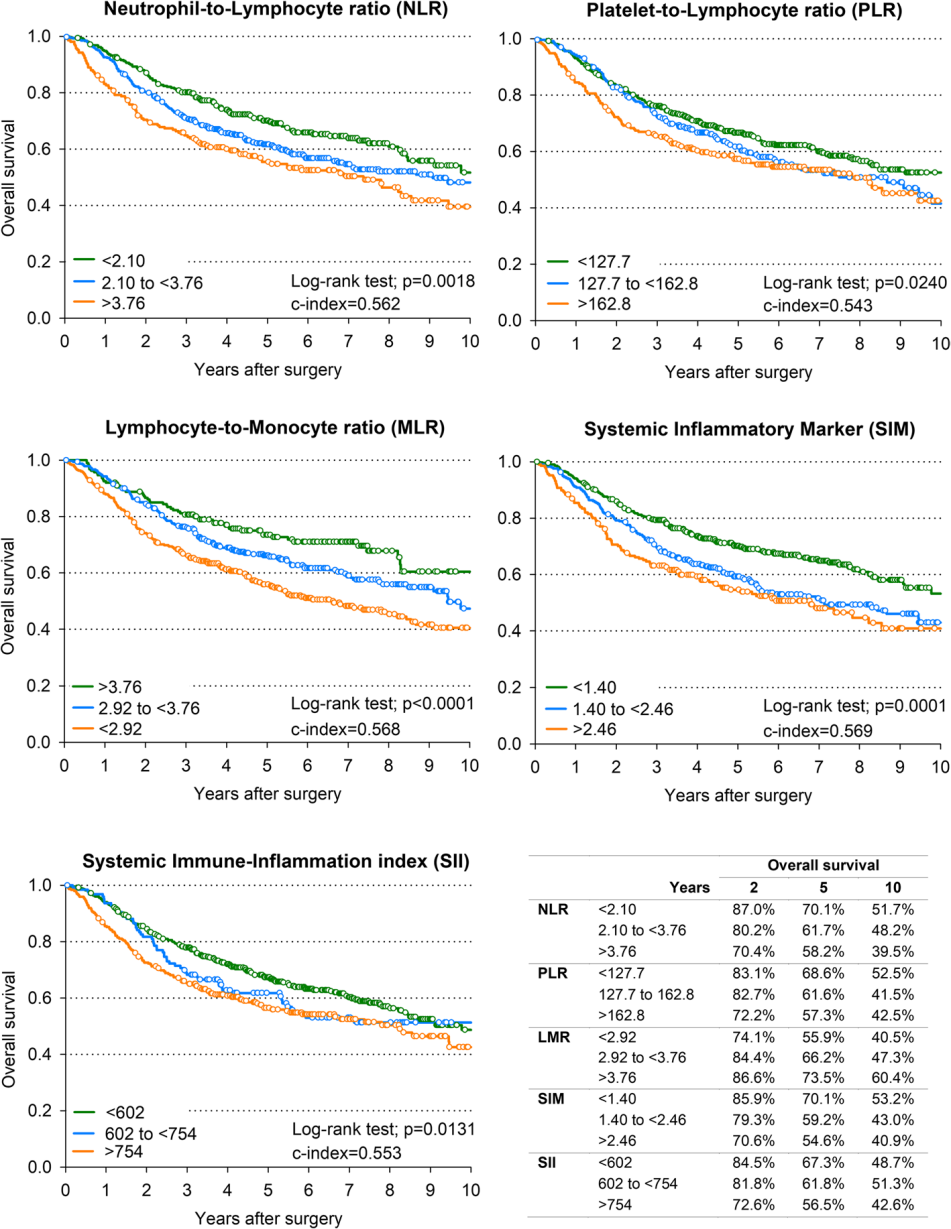
Overall survival according to inflammatory blood markers. Kaplan–Meier estimates of overall survival according to level of neutrophil-to-lymphocyte ratio, platelet-to-lymphocyte ratio, lymphocyte-to-monocyte ratio, systemic inflammatory marker, and systemic immune-inflammation index

**Fig. 2:**
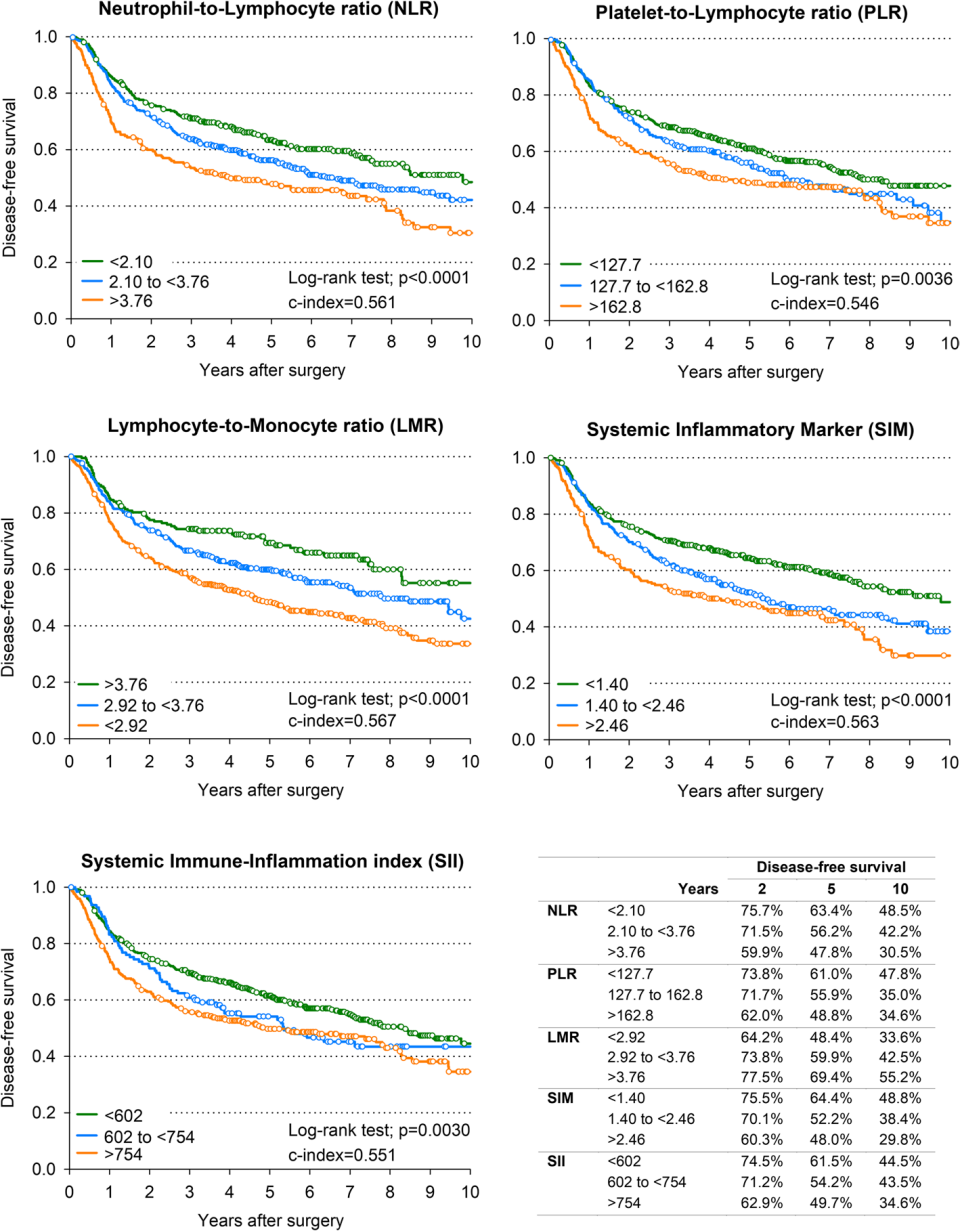
Disease**-**free survival according to inflammatory blood markers. Kaplan–Meier estimates of disease-free survival according to level of neutrophil-to-lymphocyte ratio, platelet-to-lymphocyte ratio, lymphocyte-to-monocyte ratio, systemic inflammatory marker, and systemic immune-inflammation index

Figure 1 shows Kaplan–Meier estimates of OS. Although all five indexes were significantly associated with outcome, the highest accuracy in predicting patients’ OS was found for SIM (10-year OS = 53.2% for SIM < 1.40 and 40.9% for SIM ≥ 2.46; c-index = 0.569) and LMR (10-year OS = 60.4% for LMR ≥ 3.76 and 40.5% for LMR < 2.92; c-index = 0.568). Similarly, LMR (10-year DFS = 55.2% for LMR ≥ 3.76 and 33.6% for LMR < 2.92; c-index = 0.567) and SIM (10-year DFS = 48.8% for SIM < 1.40 and 29.8% for SIM ≥ 2.46; c-index = 0.563) were the best predictors of DFS (Fig. 2). Table 2 shows the correlations between inflammatory indexes and patient’s outcomes. All five IBMs were significant predictors of DFS, with worse DFS for NLR ≥ 3.76 (HR = 1.47, 95% CI: 1.12–1.92), PLR ≥ 162.8 (HR = 1.35, 95% CI: 1.07–1.71), LRM < 2.92 (HR = 1.58, 95% CI: 1.18–2.12), SIM ≥ 2.46 (HR = 1.48, 95% CI: 1.15–1.91) and SII ≥ 754 (HR = 1.37, 95% CI: 1.11–1.70). Associations with OS were slightly weaker.

**Table 2:** Risk of local failure, regional failure, distant failure, recurrence, and death according to levels of inflammatory blood markers

	Pts	Local failure		Regional failure		Distant failure		Recurrence/Death		Death	
		HR (95% CI)^a^	Wald $\chi$^2^	HR (95% CI)^a^	Wald $\chi$^2^	HR (95% CI)^a^	Wald $\chi$^2^	HR (95% CI)	Wald $\chi$^2^	HR (95% CI)	Wald $\chi$^2^
Neutrophil-to-lymphocyte ratio (NLR)											
< 2.10	311	Ref		Ref		Ref		Ref		Ref	
2.10 to < 3.76	406	0.97 (0.64–1.47)	*p* = 0.8815	1.76 (1.12–2.75)	*p* = 0.0139	1.14 (0.71–1.86)	*p* = 0.5813	1.05 (0.83–1.34)	*p* = 0.6739	1.05 (0.81–1.36)	*p* = 0.7063
≥ 3.76	208	1.15 (0.71–1.87)	*p* = 0.5733	1.82 (1.06–3.14)	*p* = 0.0303	1.37 (0.78–2.40)	*p* = 0.2710	1.47 (1.12–1.92)	*p* = 0.0048	1.39 (1.05–1.85)	*p* = 0.0231
Platelet-to-lymphocyte ratio (PLR)											
< 127.7	465	Ref		Ref		Ref		Ref		Ref	
127.7 to < 162.8	228	0.87 (0.56–1.34)	*p* = 0.5267	1.52 (0.95–2.44)	*p* = 0.0808	0.83 (0.48–1.44)	*p* = 0.5089	1.15 (0.90–1.45)	*p* = 0.2629	1.17 (0.91–1.51)	*p* = 0.2251
≥ 162.8	232	1.44 (0.97–2.13)	*p* = 0.0718	1.98 (1.24–3.15)	*p* = 0.0043	1.67 (1.08–2.58)	*p* = 0.0208	1.35 (1.07–1.71)	*p* = 0.0111	1.30 (1.01–1.68)	*p* = 0.0413
Lymphocyte-to-monocyte ratio (LMR)^b^											
≥ 4.28	188	Ref		Ref		Ref		Ref		Ref	
2.92 to < 4.28	331	1.57 (0.87–2.86)	*p* = 0.1363	1.55 (0.86–2.81)	*p* = 0.1488	1.17 (0.62–2.19)	*p* = 0.6348	1.24 (0.92–1.68)	*p* = 0.1643	1.20 (0.87–1.67)	*p* = 0.2667
< 2.92	405	2.16 (1.22–3.84)	*p* = 0.0087	1.89 (1.10–3.25)	*p* = 0.0220	1.44 (0.79–2.65)	*p* = 0.2348	1.58 (1.18–2.12)	*p* = 0.0021	1.52 (1.11–2.09)	*p* = 0.0098
Systemic inflammatory marker (SIM)^b^											
< 1.40	423	Ref		Ref		Ref		Ref		Ref	
1.40 to < 2.46	295	1.00 (0.65–1.56)	*p* = 0.9861	1.32 (0.86–2.01)	*p* = 0.2046	1.60 (1.02–2.50)	*p* = 0.0400	1.14 (0.90–1.44)	*p* = 0.2727	1.20 (0.94–1.54)	*p* = 0.1463
≥ 2.46	206	1.42 (0.90–2.23)	*p* = 0.1277	1.57 (0.97–2.52)	*p* = 0.0655	1.26 (0.72–2.21)	*p* = 0.4160	1.48 (1.15–1.91)	*p* = 0.0024	1.43 (1.09–1.89)	*p* = 0.0099
Systemic immune-inflammation index (SII)											
< 602	501	Ref		Ref		Ref		Ref		Ref	
602 to < 754	127	0.83 (0.49–1.42)	*p* = 0.5027	1.24 (0.72–2.14)	*p* = 0.4404	1.01 (0.55–1.86)	*p* = 0.9706	0.98 (0.74–1.31)	*p* = 0.8913	0.97 (0.71–1.32)	*p* = 0.8480
≥ 754	297	1.23 (0.85–1.79)	*p* = 0.2786	1.66 (1.10–2.51)	*p* = 0.0157	1.33 (0.87–2.02)	*p* = 0.1887	1.37 (1.11–1.70)	*p* = 0.0034	1.32 (1.05–1.66)	*p* = 0.0171

Hazard ratio (*HR*) and corresponding 95% confidence interval (*CI*) were estimated through Cox proportional hazard model, adjusting for study centre, gender, age, cancer site, pT, pN, surgical margins, extranodal extension, and adjuvant (chemo) radiotherapy. Optimal biomarkers’ cut-offs were determined through iterative procedure which maximize predictability on overall survival

^a^Adjusted for competing risk according to Fine and Gray model. ^b^One patients has missing data

**Fig. 3:**
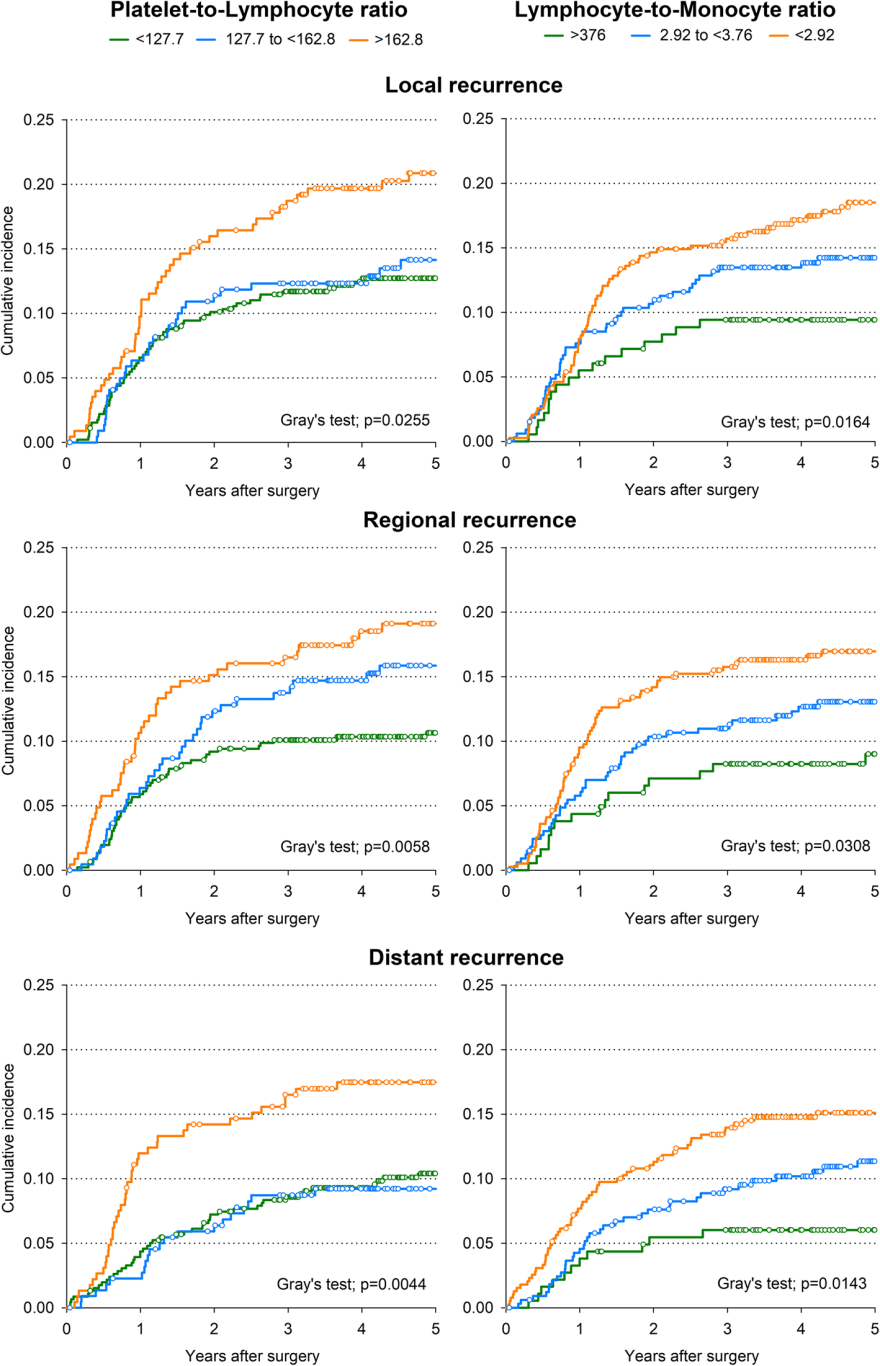
Recurrence according to inflammatory blood markers. Cumulative incidence estimates of local, regional, and distant recurrence according to level of platelet-to-lymphocyte ratio and lymphocyte-to-monocyte. Competing risk of death was accounted according to Fine-Gray method

A LMR of 2.92 or less showed the strongest association with local failure (HR = 2.16; 95% CI: 1.22–3.84), while a PLR of 162.8 or higher showed the strongest association with regional (HR = 1.98; 95% CI: 1.24–3.15) and distant failure (HR = 1.67; 95% CI: 1.08–2.58) (Table 2). Compared to patients with PLR < 127.7, those with PLR ≥ 162.8 suffered a higher 5-year cumulative incidence of local failure (20.9% versus 12.7%; *p* = 0.0255 – Fig. 3) and regional failure (19.1% versus 10.6%; *p* = 0.0058). Similar trends were found for LMR < 2.92 versus LMR ≥ 3.76 for both local failure (5-year cumulative incidence: 18.5% versus 9.4%; *p* = 0.0164) and regional failure (17.0% versus 9.0%; *p* = 0.0308).

## Discussion

The present study supports the use of five IBMs, which can be easily calculated in routine clinical practice using standard blood tests, as prognostic markers, offering evidence of a strong correlation with prognosis. All five IBMs analysed in the present study were good predictors of OS or DFS. However, LMR and SIM emerged as the two best performing indices in identifying patients at greatest risk of death. Of interest, it was also observed that different IBMs might predict specific patterns of failure. In particular, a low LMR showed the strongest association with local failure, while a high PLR showed the strongest association with regional and distant failure.

Chronic inflammation is a well-recognized tumour-enabling capability, which can promote cancer development and progression [22]. Inflammatory cells and their mediators are in fact an essential component of the tumour microenvironment (TME) with cancer cells being able to induce inflammatory reactions through various mechanisms [23]. In addition, there is significant dialogue between the mediators and cytokines in the local TME and the peripheral circulating compartment [24]. The tumour-derived secretome can indeed influence bone marrow to increase myelopoiesis resulting in a change in the proportions of neutrophils, monocytes, platelets, and lymphocytes [25]. Thus, IBMs based on peripheral blood-based parameters have been studied as surrogate biomarkers that may capture the inflammatory cross-talk between cancer and immune cells in the local TME [26].

Although we cannot provide a mechanistic explanation for our observations, these results are consistent with the recent evidence emerging about the role played by different inflammatory cells of the TME. While lymphocyte activation can be counterbalanced by a broad spectrum of immunosuppressive mechanisms that are present in the TME, including programmed cell death protein‐1 (PD‐1) and Forkhead box P3 (FOXP3) CD4 + regulatory T-cells [27], the presence of tumour infiltrating lymphocytes (TILs) in the TME is a positive prognostic marker in multiple solid tumours [28]. In particular, CD4 + T-helper 1 (Th1) cells facilitate antigen presentation through cytokine secretion and activation of antigen presenting cells, while CD8 + cytotoxic T-cells (CTL) are essential for tumour destruction [29]. On the other hand, tumour-infiltrating B-lymphocytes suppress tumour progression by secreting immunoglobulins, promoting T-cell response, and killing cancer cells directly. Finally, NK cells are part of the innate immune system able to kill tumour cells and prevent metastasis [30]. An immunohistochemical investigation showed that patients with pharyngeal and laryngeal cancer, whose tumours were densely infiltrated by T cells, cytotoxic T cells, NK cells, and stromal dendritic cells, had an improved outcome compared to patients whose cancers were poorly infiltrated [31]. Consistently, the results from a recent meta-analysis demonstrated the positive prognostic significance of CD8 + and CD4 + TILs in HNSCC [32].

Moreover, several studies have observed an inverse correlation between the NLR and the number of TILs in the TME, with a low NLR correlating with high levels of tumour-infiltrating CD8 + T cells [33, 34], thus supporting the concept that the peripheral blood compartment may predict the immune milieu in the TME.

As commonly available and measurable an index, LNR has been widely investigated as a potential prognostic biomarker in oncology. In a recent and very large retrospective series, it was observed that HNSCC patients with high NLR had both an increased hazard of all-cause mortality and of cancer-specific mortality [35]. Interestingly, baseline NLR > 5 was associated with poor outcome in patients with recurrent or metastatic HNSCC treated with nivolumab [36]. Moreover, a high NLR was significantly associated with cancer cachexia development [37] and was shown to be an independent predictor of mortality in HNSCC patients treated with primary or adjuvant chemoradiation [38]. Consistently with the above observation, NLR was found to be an independent predictor of both DFS and OS also in the present surgical series.

Macrophages are the most abundant leukocytes in the TME. Tumour-associated macrophages (TAMs) are drivers of tumour progression in established tumours, promoting cancer cell proliferation and survival, angiogenesis and lympho-angiogenesis, reducing effective T-cell responses [39]. Circulating monocytes give rise to macrophages that reside in tissues. Importantly, macrophage differentiation from monocytes occurs in the peripheral tissue in association with the gaining of a functional phenotype that depends on microenvironmental signals. Particularly, tumour and stromal cells release chemotactic factors, i.e. chemokines ligand-2 and -5, that recruit macrophages and contribute to macrophage polarization toward specific phenotypes, with interleukin (IL)-4, IL-13, IL-23, immunocomplexes, transforming growth factor (TGF)-β, or macrophage colony-stimulating factor (M-CSF) diverting macrophage polarization through an M2/M2-like phenotype, which sustains many aspects of tumour growth and progression [40]. Myeloid-derived suppressor cells (MDSCs), a heterogeneous population of cells, which can suppress T-cell responses and expand during inflammation and cancer, are also derived from monocytes. Interestingly and consistently with our observation that a low LMR showed the strongest association with local failure, tumour associated macrophages (TAMs) and MDSCs may promote field cancerization by generating reactive oxygen species (ROS) that create an immunosuppressive environment allowing adjacent precancerous cells to grow, expand, and acquire a fully malignant phenotype [41]. However, no studies conducted in patients with HNSCC have investigated the predictive value of LMR specifically for local recurrence. Several studies conducted in patients with cancers other than HNSCC have reported a significant association between a low LMR and the risk of recurrence without specifying the type of tumour relapse [42–44]. Furthermore, TAMs have also been implicated in tumour invasion and metastasis through production of proteases, which digest the components of the extracellular matrix, by promoting the angiogenic switch and sustaining lymph angiogenesis. This may explain the weaker but still significant association between a low LMR and the risk of developing lymph node metastases observed in this series of patients.

Platelets contribute significantly to the metastatic process [45, 46]. Platelets form a shield around metastatic cells protecting them from immune surveillance, and also induce matrix metalloproteinase (MMP)-9 expression and activation leading to increased remodelling of the extracellular matrix, release of growth factors from the extracellular matrix, and relief of intercellular contacts, thus facilitating cancer cell dissemination and metastasis [45]. Finally, platelets can also increase microvascular permeability, leading to intravasation of cancer cells into the circulation [46].

These properties may explain the association we observed in our series between a high PLR and a significantly higher risk of regional and distant failure. In previous work on HNSCC, PLR has mainly been investigated for its ability to predict OS and DFS but not more specific endpoints [47–49]. However, preoperative PLR has been observed to be superior to NLR as a predictive marker for lymph node metastasis in oral squamous cell carcinoma [50]. Similarly, a recent meta-analysis showed that a high PLR was significantly associated with a higher risk of lymph node metastasis in patients with gastric cancer [51]. Moreover, in breast cancer, a persistently high PLR was observed to be associated with worse metastatic-free survival [52]. These findings support the hypothesis that PLR may be a surrogate marker for the propensity of cancer cells to metastasize. Of interest, PLR was also found to be a predictor of good response to induction chemotherapy in patients with HNSCC [53].

IBMs have been investigated in HNSCC mainly in single-centre retrospective studies. The present multicentre study provides a large cohort of highly selected patients with strict inclusion criteria, and follow-up with regular clinical examination as recommended by the American Cancer Society. However, this study has some limitations. Firstly, the retrospective design may have biased the results. Secondly, blood parameters were collected pre-operatively in order to avoid the influence of surgery itself on the baseline values. However, it was not always possible to exclude the effect of any other systemic condition, as the investigated IBMs are not tumour-specific markers. Furthermore, specific treatment protocols (including the type of surgery) were not assessed, therefore, the estimated risk did not account for them. However, multivariable model included the study centre as a covariate, which may have partially captured the heterogeneity of treatment approaches across centres. Lastly, given the period of time during which selected patients were diagnosed and treated, HNSCC were staged according to the 7^th^ edition of the American Joint Commission on Cancer TNM. However, considering that we excluded patients with HPV-driven cancers, the divergence between the 7^th^ and the 8^th^ editions of TNM in our series is limited.

## Conclusions

In this retrospective observational study of patients undergoing surgical treatment for HPV-negative HNSCC, different IBMs were observed to be predictive of different patterns of failure. Thus, different IBMs may be useful not only to stratify prognosis but also to identify patients at risk of local, regional, and distant recurrences thus guiding surveillance follow-up strategies and (neo)adjuvant treatment. In this regard, the integration of immunotherapy as therapeutic weapon in the comprehensive approach to HNSCC may be better tailored according to the above-described parameters. Further research is required to confirm and validate these findings, to investigate their use in non-surgically managed HNSCC, and to support the use of different IBMs to predict different clinical endpoints.

## Supplementary Information

**Additional file 1: Table S1. **Risk of recurrence and death according to blood parameters

## Data Availability

The datasets generated during and/or analysed during the current study are available from the corresponding author on reasonable request.

## Abbreviations

CI: Confidence interval
DFS: Disease-free survival
HNSCC: Head and neck squamous cell carcinoma
HR: Hazard ratio
IBM: Inflammatory blood markers
LMR: Lymphocyte to monocyte ratio
MDSC: Myeloid-derived suppressor cell
NLR: Neutrophil to lymphocyte ratio
OS: Overall survival
PLR: Platelet to lymphocyte ratio
SII: Systemic immune-inflammation index
SIM: Systemic inflammatory marker
TAM: Tumour-associated macrophage
TIL: Tumour infiltrating lymphocytes
TME: Tumour microenvironment
