# Supplementary material for: Different inflammatory blood markers correlate with specific outcomes in incident HPV-negative head and neck squamous cell carcinoma: a retrospective cohort study
Source: BMC Cancer. 2022 Mar 5;22:243. doi: 10.1186/s12885-022-09327-4 (PMC8897882; doi:10.1186/s12885-022-09327-4)
Supplement: Supplementary file 1 — Additional file 1: Table S1. Risk of recurrence and death according to blood parameters [file 12885_2022_9327_MOESM1_ESM.docx]

**Supplementary Table 1**. Risk of recurrence and death according to blood parameters.

|  | **Patients** | **Disease-free survival** | | |  | **Overall survival** | | |
| --- | --- | --- | --- | --- | --- | --- | --- | --- |
|  |  | **Events** | (%) | **HR (95% CI)** |  | **Events** | (%) | **HR (95% CI)** |
| Haemoglobin^a^ |  |  |  |  |  |  |  |  |
| Normal/High | 628 | 271 | (43.2) | Ref. |  | 224 | (35.7) | Ref. |
| Low | 297 | 175 | (58.9) | 1.35 (1.10-1.68) |  | 161 | (54.2) | 1.56 (1.24-1.95) |
| Lymphocytes^b^ |  |  |  |  |  |  |  |  |
| ≥1.97 | 383 | 157 | (41.0) | Ref. |  | 136 | (35.5) | Ref. |
| 1.44 to <1.97 | 309 | 162 | (52.4) | 1.37 (1.09-1.72) |  | 142 | (46.0) | 1.38 (1.08-1.77) |
| <1.44 | 233 | 127 | (54.5) | 1.34 (1.05-1.72) |  | 107 | (45.9) | 1.33 (1.02-1.73) |
| Monocytes^b^ |  |  |  |  |  |  |  |  |
| <0.59 | 446 | 203 | (45.5) | Ref. |  | 172 | (38.6) | Ref. |
| 0.59 to <0.78 | 241 | 114 | (47.3) | 1.11 (0.88-1.41) |  | 104 | (43.2) | 1.21 (0.94-1.56) |
| ≥0.78 | 237 | 128 | (54.0) | 1.14 (0.90-1.46) |  | 108 | (45.6) | 1.06 (0.81-1.38) |
| Neutrophyls^b^ |  |  |  |  |  |  |  |  |
| <4.28 | 377 | 161 | (42.7) | Ref. |  | 137 | (36.3) | Ref. |
| 4.28 to <5.54 | 245 | 140 | (57.1) | 1.25 (0.98-1.58) |  | 123 | (50.2) | 1.28 (0.99-1.65) |
| ≥5.54 | 303 | 145 | (47.9) | 1.06 (0.83-1.35) |  | 125 | (41.3) | 1.04 (0.80-1.36) |
| Platelets^b^ |  |  |  |  |  |  |  |  |
| <160 | 150 | 87 | (58.0) | 1.33 (0.98-1.79) |  | 77 | (51.3) | 1.32 (0.95-1.84) |
| 160 to <236 | 350 | 149 | (42.6) | Ref. |  | 127 | (36.3) | Ref. |
| ≥236 | 425 | 210 | (49.4) | 1.24 (1.01-1.53) |  | 181 | (42.6) | 1.23 (0.98-1.54) |

Hazard ratio (HR) of recurrence and death and corresponding 95% confidence intervals (CI) were estimated through

Cox proportional hazard model, adjusting for study centre, gender, age, cancer site, pT, pN, surgical margins, extranodal extension, and adjuvant (chemo)radiotherapy. ^a^Haemoglobin level was define as low when <12 g/dL in women and <14 g/dL in men; normal when in 12-16 g/dL in women and in 14-18 g/dL in men; high otherwise. Only 10 patients reported high haemoglobin level.^b^Optimal cut-offs were determined through iterative procedure which maximize predictability on overall survival.
